# OPTIMUM: a protocol for a multicentre randomised controlled trial comparing Out Patient Talc slurry via Indwelling pleural catheter for Malignant pleural effusion vs Usual inpatient Management

**DOI:** 10.1136/bmjopen-2016-012795

**Published:** 2016-10-18

**Authors:** P Sivakumar, A Douiri, A West, D Rao, G Warwick, T Chen, L Ahmed

**Affiliations:** 1 St Thomas’ Hospital, London, UK; 2 King's College London, London, UK; 3 Princess Royal University Hospital, Orpington, UK; 4 King's College Hospital, London, UK

**Keywords:** Malignant pleural effusion, Quality of life, Indwelling pleural catheter

## Abstract

**Introduction:**

The development of malignant pleural effusion (MPE) results in disabling breathlessness, pain and reduced physical capability with treatment a palliative strategy. Ambulatory management of MPE has the potential to improve quality of life (QoL). The OPTIMUM trial is designed to determine whether full outpatient management of MPE with an indwelling pleural catheter (IPC) and pleurodesis improves QoL compared with traditional inpatient care with a chest drain and talc pleurodesis. OPTIMUM is currently open for any centres interested in collaborating in this study.

**Methods and analysis:**

OPTIMUM is a multicentre non-blinded randomised controlled trial. Patients with a diagnosis of MPE will be identified and screened for eligibility. Consenting participants will be randomised 1:1 either to an outpatient ambulatory pathway using IPCs and talc pleurodesis or standard inpatient treatment with chest drain and talc pleurodesis as per British Thoracic Society guidelines. The primary outcome measure is global health-related QoL at 30 days measured using the EORTC QLQ-C30 questionnaire. Secondary outcome measures include breathlessness and pain measured using a 100 mm Visual Analogue Scale and health-related QoL at 60 and 90 days. A sample size of 142 patients is needed to demonstrate a clinically significant difference of 8 points in global health status at 30 days, for an 80% power and a 5% significance level.

**Ethics and dissemination:**

The study has been approved by the NRES Committee South East Coast—Brighton and Sussex (reference 15/LO/1018). The trial results will be published in peer-reviewed journals and presented at scientific conferences.

**Trial registration numbers:**

UKCRN19615 and ISRCTN15503522; Pre-results.

**Strengths and limitations of this study**

- This is the first prospective randomised controlled trial primarily comparing quality of life (QoL) outcomes between inpatient and outpatient treatment pathways for malignant pleural effusion.
- The primary outcome measure is clinically relevant.
- Patients with non-expandable lung are also included in the study.
- The study represents real life presentation and management.
- A limitation is that patients with poor performance status are not included and only patients with potential for ambulatory management are included. However, to include those patient groups a separate study design to answer that specific question will have to be conducted.

## Introduction

Malignant pleural effusion (MPE) is a common presentation in advanced malignancy, complicating nearly 50% of all lung and breast cancers.^1^ Extrapolated data estimate 50 000 new cases of MPE per year in the UK, translating to one new case per 1000 population per year.^2^ MPE results in significant morbidity, including disabling breathlessness, pain and reduced physical capability. With advancement of techniques over the last decade, physicians and patients are faced with a number of choices in the management of MPE. These choices depend on many factors, and not all patients are suitable for every strategy. Regardless of the choice, the aim should be an overall improvement in quality of life (QoL).

Managing patients with an easily implemented ambulatory pathway which averts the need for an inpatient hospital stay may improve QoL in a population with an otherwise poor prognosis.^3^ To investigate this, the OPTIMUM trial, a National Institute for Health Research (NIHR) Portfolio multicentre study (UKCRN 19615), is currently open to taking on other recruiting sites.

### British Thoracic Society guidelines

The 2010 British Thoracic Society (BTS) pleural disease guideline advocates chest tube drainage and chemical pleurodesis involving a hospital admission.^4^ The sclerosant of choice in the UK is graded large-particle talc, a safe and effective sclerosant at a dose of 4 g.^4^ Median length of a hospital admission for this intervention is 4 days (IQR 2–6 days).^5^

### The indwelling pleural catheter

An alternative and increasingly popular strategy to manage MPE is the insertion of an indwelling pleural catheter (IPC) as an outpatient and continued management on an outpatient/ambulatory basis. The BTS recognises that IPCs are effective in controlling recurrent effusions and are particularly useful in cases of trapped lung, where talc pleurodesis is likely to fail. It is a safe procedure which can be managed in the outpatient setting, thus avoiding hospital admission.^6^

A relatively new treatment pathway involves instillation of talc via an IPC to prevent recurrence, allowing the opportunity to manage people as outpatients. This novel pathway in the management of MPE is well established at Guy's and St Thomas's Hospital. Our case series of 24 patients who underwent talc pleurodesis via an IPC confirms the safety and efficacy of this treatment algorithm^7^ on which the OPTIMUM trial is based.

A huge body of research has looked into the therapeutic management of MPE. This has led to multiple management pathways. However, there is no one definitive management strategy. Despite the goal of palliation, the majority of studies focus on outcome measures such as pleurodesis success rate, failure rate and complications.^6^ ^8–12^ While this is important, we believe it is important to understand the impact of these interventions on the overall well-being of patients.

There is a paucity of data on QoL outcomes in this population, particularly the effects of different pleural interventions. A retrospective study of talc pleurodesis by surgical video-assisted thoracoscopy failed to show any QoL benefit at 3 and 6 months.^13^ The Second Therapeutic Intervention in Malignant Effusion (TIME2) randomised controlled trial showed no difference between medical pleurodesis and IPC insertion without talc in relieving breathlessness. Global QoL evaluated as a secondary measure did improve in both groups at 6 weeks, but there was no significant difference in QoL at any time point.^5^ There are no prospective randomised controlled trials primarily comparing QoL outcomes in the outpatient and inpatient management of MPE.

## Objectives

### Hypothesis

The primary hypothesis is that outpatient management of MPEs with an IPC improves global health-related QoL at 30 days following insertion when compared to standard treatment according to BTS guidelines.

With no validated QoL assessment tools for patients with MPE, global health-related QoL as measured by the EORTC QLQ-C30 has been selected as the primary outcome measure. It is an extensively used tool and validated for malignant disease of various histological types.^14^ Using one tool to assess health-related QoL will ensure that the protocol limits the burden to trial participants.

Primary and secondary objectives and outcome measures are summarised in table 1.

**Table 1:** Primary and secondary objectives for the OPTIMUM trial

Objectives	Outcome measures	Time points of evaluation
*Primary objective*		
To assess whether a minimally invasive evidence-based pathway for the outpatient management of malignant pleural effusion improves global health-related quality of life at 30 days	Self-reported health-related quality of life based on EORTC QLQ-C30 questionnaire	Day 30
*Secondary objectives*		
Improvement in global health-related quality of life at 60 and 90 days	Self-reported health-related quality of life based on EORTC QLQ-C30 questionnaire	Day 60, 90
Pleurodesis failure rate	Subsequent pleural intervention required on the same side as pleurodesis Chest X-ray opacification greater than 25% on side of intervention judged by two independent clinicians	Day 30, 60, 90
Improvement in symptoms of pain and breathlessness	Medical Research Council (MRC) Dyspnoea Scale 100 mm Visual Analogue Scale for pain and breathlessness	Day 30, 60 and 90
Complication rate	Clinical review and adverse event documentation	Day 7, 14, 30, 60 and 90

## Methods and analysis

### Trial design

The OPTIMUM trial is a prospective, two-arm non-blinded randomised multicentre trial. The Medicines and Healthcare products Regulatory Agency have cleared this trial as a non-clinical trial of investigational medicinal product (non-CTIMP) study. All patients presenting to recruitment centres with MPEs who fulfil the inclusion criteria will be approached. The trial is currently open at eight sites in the UK: St Thomas' Hospital, London; King's College Hospital, London; Princess Royal Hospital, Bromley; East Surrey Hospital, Redhill; East Sussex Hospital, Eastbourne; St George's Hospital, London; Hull Royal Infirmary, Hull and Medway Maritime Hospital, Gillingham. At the time of publication, we are actively seeking more sites to recruit.

### Study population

A total of 142 patients in participating respiratory departments meeting the eligibility criteria will be recruited. All sites have a dedicated pleural service with experience in IPC insertion.

### Inclusion criteria

1. Diagnosis of MPE.^i^
2. WHO performance status 2 or less unless performance status is impaired by the presence of effusion and likely to significantly improve with drainage.
3. Expected survival <3 months.

### Exclusion criteria

1. Age <18 years old.
2. Pregnant or lactating.
3. Known allergy to talc or lignocaine.
4. Lack of symptomatic relief from effusion drainage.
5. District nurse/carers/hospital team unable to carry out at least twice weekly drainage from an IPC.
6. Lymphoma or small cell carcinoma except:^ii^
  - Failure of chemotherapy.
  - Deemed for palliative management.
7. Non-malignant effusions.
8. Loculated pleural effusion that would prevent successful drain insertion and clinical benefit to the patient.
9. Unable to provide written informed consent to trial participation.

### Recruitment and randomisation

Enrolment started in July 2015 and is anticipated to continue until January 2020. In each centre, the trial team will screen patients presenting to the respiratory and oncology clinics as well as inpatient wards.

Once screening procedures have confirmed a patient's eligibility, they will be approached and consented for the study. They will undergo stratified randomisation (1:1) to either usual care as per BTS guidance or treatment with an IPC. This will be performed using a web-based secure randomisation service for clinical trials (http://www.sealedenvelope.com). The stratification variables are age (<65 years, ≥65 years), malignancy subtype (lung, mesothelioma, breast, other) and WHO performance status (0, 1, 2, 3). Given the nature of the intervention, patients and the research team will not be blinded.

### Study procedure

Table 2 outlines the schedule of enrolment, interventions and assessments. Patients will undergo baseline chest X-ray and ultrasound assessment. QoL and symptom data will be collected using the EORTC QLQ-C30 questionnaire, MRC Dyspnoea Scale and 100 mm Visual Analogue Score (VAS) for pain and breathlessness. These questionnaires will be repeated for both groups during each follow-up visit.

**Table 2:** Schedule of enrolment, interventions and assessments

	Both groups	Usual care group					IPC group					
Study procedures	Consent/baseline	Postrandomisation	Days 2–5	Day 7	Day 14	Day 30, 60, 90	Postrandomisation	Day 4	Day 7	Day 14	Day 30, 60, 90	Ongoing (between follow-up)
Sign consent	X											
Demographics/medical history/blood pressure	X											
Randomisation	X											
Local anaesthesia		X					X					
Ultrasound-guided IPC or chest drain insertion		X					X					
Pleural manometry		X					X					
Daily observations (HR, respiratory rate, oxygen requirement, BP, chest drain assessment) drain in situ			X									
Inpatient drainage			X									
Community drainages												X
Drainage booklet							X					X
Pleurodesis			X					X				
Drain removal			X							X		
AE data collection		X	X	X	X	X	X	X	X	X	X	X
Chest X-ray	X	X	X	X	X	X	X	X*	X	X	X	X
Thoracic ultrasound	X			X	X	X		X	X	X	X	
VAS assessment (pain and breathlessness)	X			X	X	X			X	X	X	
EORTC QLQ -C30	X			X	X	X			X	X	X	
MRC Dyspnoea Score	X			X	X	X			X	X	X	
WHO performance status	X											

*Optional CXR based on USS appearances.

AE, adverse events; CXR, chest X-ray; IPC, indwelling pleural catheter; MRC, Medical Research Council; USS, ultrasound scan.

### The indwelling pleural catheter group

**Figure 1:**
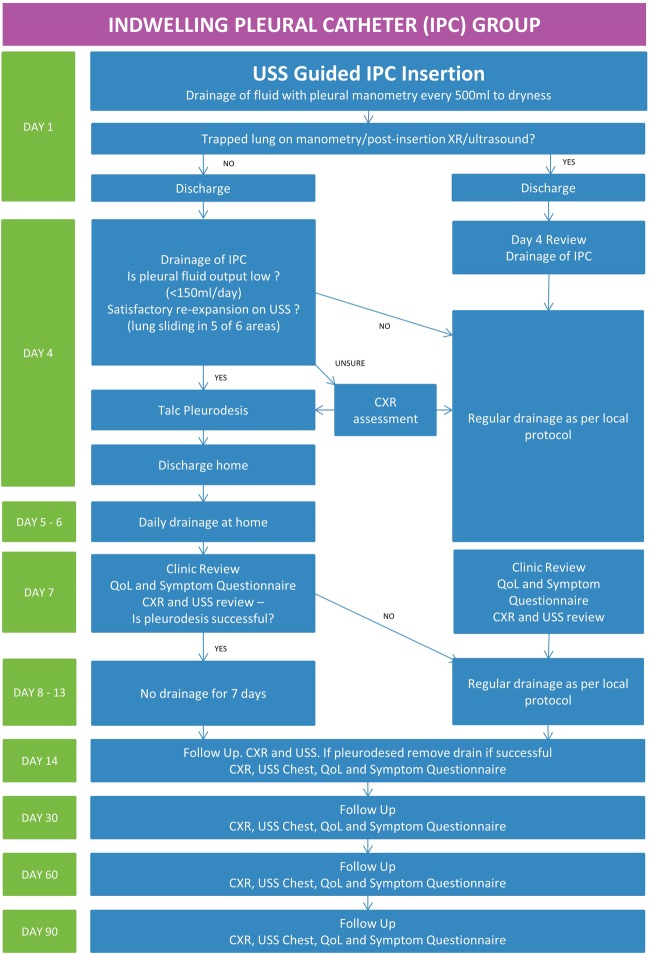
Trial pathway for the indwelling pleural catheter group. CXR, Chest X-ray; USS, ultrasound scan; IPC, indwelling pleural catheter; QoL, quality of life.

The IPC intervention algorithm is summarised in figure 1. Patients allocated to the IPC group will undergo chest ultrasound to assess the pleura, pleural fluid and to identify a safe site for insertion.

The IPC will be inserted as per normal practice.^9^ Once the indwelling catheter is inserted, intrapleural pressure will be measured with a water manometer (Medifix Manometer Scale, B. Braun, Sheffield, UK), and an attempt will be made to evacuate as much fluid as is tolerated while monitoring the patient's symptoms and oxygen saturation. Serial measurements of pleural pressure will be recorded for every 500 mL of fluid drained continuing drainage until one of three criteria is met:

1. Effusion fully drained.
2. Patient symptomatic with pain, cough or presyncope.
3. End expiratory pleural pressure drops below 20 cm H_2_O or pleural elastance >10 (pleural elastance is calculated by dividing the change in pleural pressure by the volume (in litres) removed).

A postinsertion chest X-ray will be performed to confirm the position of the catheter. Patients will be discharged, and a district nurse referral completed if required. Patients on steroids will be asked, if clinically possible, to discontinue steroids 48 hours prior to their visit on day 4 given the potential for talc slurry. In steroid-dependent patients, admission to hospital prior to pleurodesis will be at the discretion of the clinician as per standard practice.

Patients will be reviewed on day 4 (±24 hours) postprocedure. The pleural fluid will be drained via the IPC. The quantity of fluid removed, and re-expansion of lung will be assessed. If the average pleural fluid output is low (<150 mL/day) and if satisfactory re-expansion of lung is confirmed by ultrasound, talc pleurodesis will be attempted through the IPC.

**Figure 2:**
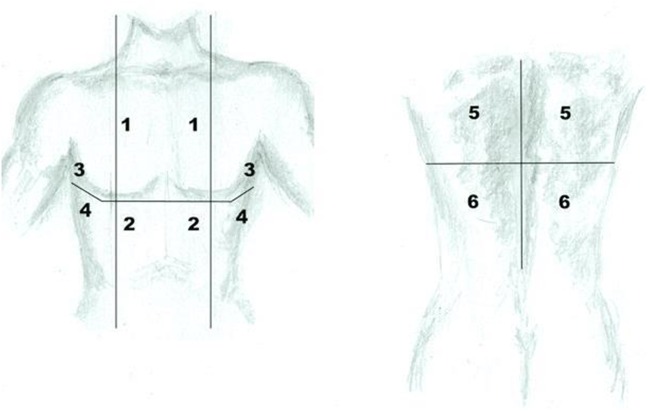
Sites of thoracic ultrasound to assess for pleural apposition.

Satisfactory lung re-expansion will be defined as lung sliding on the chest wall in at least five of six defined areas in the chest (figure 2). In the case of uncertainty, chest radiographs will be used. A diagnosis of lung entrapment can be made using the pleural manometry data, ultrasound and chest radiograph appearances. For example, if uncertainty exists over potential small areas of entrapment on ultrasound and the X-ray appearances are satisfactory, talc may be administered.

If the lung has not re-expanded, continued regular drainage (depending on fluid output) will be advised.

If talc pleurodesis is attempted based on the above criteria, this will be administered as a ‘slurry’ via the IPC. Then, 3 mg/kg (max 250 mg) 1% lignocaine will be instilled into the pleural space via the IPC to prevent any acute pain. This will be followed by 4 g of talc mixed with 50 mL normal saline via the drain followed by 50 mL normal saline flush. Patients will be observed for at least 1 hour following talc instillation.

The patient will then be sent home with the advice to drain daily with a 1 L vacuum bottle for 3 days. This can be performed by themselves, their carer or district nurse. They will be reviewed on the third day postpleurodesis (+48 hours). They will be asked to complete health-related QoL and symptom questionnaires and undergo ultrasound and chest X-ray review of the pleural collection.

If there is no recurrence of effusion with satisfactory ultrasound evidence of pleural symphysis in at least five out of six areas, patients will be advised not to undertake any drainage for further 7 days. On day 14 (±24 hours) postintervention, they will undergo QoL and symptom questionnaires, ultrasound and chest X-ray review. If pleurodesis is successful, the IPC will be removed.

Successful pleurodesis will be defined as pleural symphysis in at least five out of six areas (see figure 2) on ultrasound as evidenced by the absence of lung sliding in these areas.

Patients will undergo a clinic review at 30, 60 and 90 day's postprocedure. At each visit, a chest X-ray and chest ultrasound scan will be performed recording X-ray appearances and the presence of pleural thickening, depth of effusion and extent of septations on ultrasound. Patients will be required to complete the EORTC QLQ-C30 questionnaire, MRC Dyspnoea Scale and VAS for pain and breathlessness.

### The usual care group

**Figure 3:**
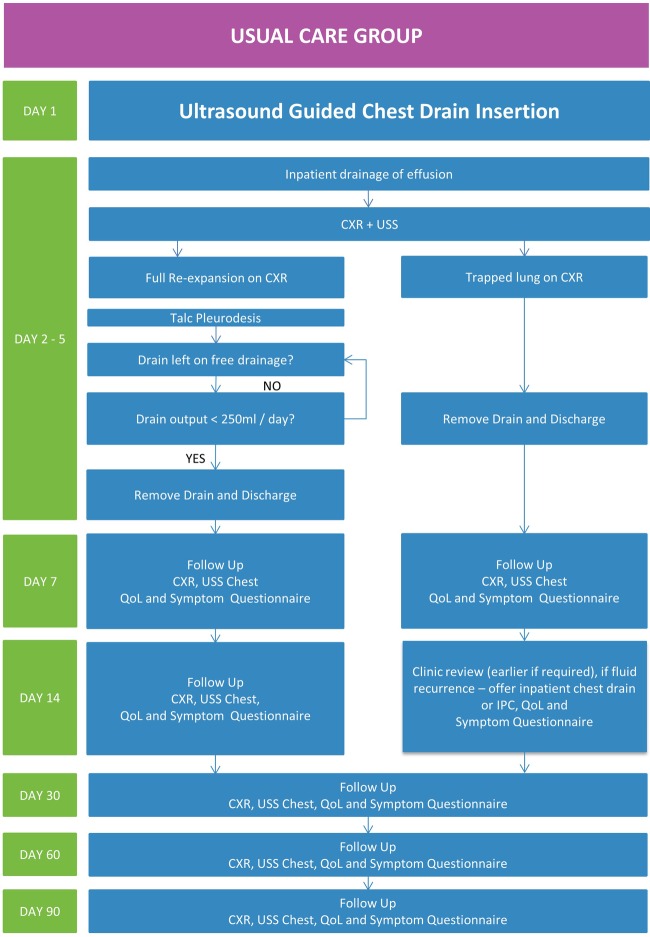
Trial pathway for the usual care group. CXR, chest X-ray; USS, ultrasound scan; IPC, indwelling pleural catheter; QoL, quality of life.

The usual care pathway is summarised in figure 3. Patients randomised to the usual care group will be managed according BTS guidelines. They will be admitted to hospital for drain insertion and undergo a thoracic ultrasound to assess the pleura, pleural fluid and to identify a safe site for insertion. A standard 10 French—14 French gauge chest drain will be inserted. Fluid will be emptied gradually measuring oxygen saturations and monitoring for symptoms. Pleural manometry will be recorded.

From day 2 to 5 patients will be assessed for complications, drain output and lung re-expansion. If clinically possible, any steroids will be discontinued. Once full lung re-expansion is confirmed on X-ray (defined as full re-expansion of the lung to the chest wall or only blunting of the costophrenic angle on the affected side), talc slurry will be performed.

If lung re-expansion is unsatisfactory with evidence of trapped lung on chest X-ray (<50% pleural apposition on chest X-ray), then pleurodesis will not be attempted and the drain removed with early follow-up of the patient to consider repeated drainage of the effusion or IPC insertion if symptomatic.

If pleurodesis is performed, it will be carried out as per the BTS guidelines. Then, 3 mg/kg (maximum 250 mg) of 1% lignocaine will be administered via the chest drain. This will be followed by 4 g talc mixed with 50 mL saline instilled through the drain, followed by 50 mL saline flush of the drain into the pleural cavity. The drain will be clamped and left closed for 1 hour. After this time, the drain will be opened and left on free drainage for 24–72 hours. If the fluid output is <250 mL per day, the drain will be removed and patient will be discharged home. Complications, drain output and the use of thoracic suction (which will be at the discretion of the treating team) will be recorded. If the output is high, the drain will be left longer and depending on the clinical assessment as per the standard practice, the drain will be removed.

Patients will have follow-up on the 7th day and 14th day postprocedure. During each follow-up, they will undergo a chest X-ray and chest ultrasound and be asked to complete the QoL and symptom questionnaires. They will also undergo the same assessment at follow-up 30, 60 and 90 days after the intervention. If the pleurodesis is not successful, they will have further assessment and will be managed based on clinical requirement regarding the need for further pleural intervention.

Patients may choose to withdraw themselves from the trial. If a patient does so, we will retain the data the patient has previously consented for us to collect and analyse. No further data would be collected. It will be made very clear to the patient throughout their follow-up that a decision to withdraw from the trial will not prejudice any future medical care they receive. On completion of the trial, patients will receive normal medical care as per local practices.

### Sample size calculation and statistical analysis

In this ANCOVA study, sample sizes of 71 and 71 are needed from each of the 2 groups whose means are to be compared. The covariate has an *R*^2^ of 0.49 (0.7 correlation between baseline and follow-up). A total sample of 142 participants achieves 80% power to detect a clinically significant difference of 8 points in global health status at 30 days among the means versus the alternative of equal means using an F test with a 5% significance level. The minimally important difference in global health status is based on reference values provided by the EORTC Quality of Life Group.^15^ The common SD within a group is assumed to be 23.60, derived from EORTC reference values (all patients with cancer: stages III and IV).^15^ Therefore, the randomisation target is 142 participants. The interim analysis is planned when 50% of the randomisation target has been reached and will include review of recruitment, follow-up rates and the randomisation target.

### Trial management

The respiratory research team at St Thomas' hospital will oversee site initiation and training, screening log and data submission, data quality assurance and study close out. The sponsor Guy's and St Thomas' Hospital R&D development will oversee contracts agreements at each site. Sites will undergo regular monitoring by the coordinating trial team (consisting of the chief investigator, trial manager and trial coordinator) through both site visits as well as the assessment of submitted data quality and adverse events (AEs). A yearly progress report will also be submitted to the Health Research Authority.

### Data management

These data will be collected by the trial coordinator, principal investigators and research nurse. Patient data will be anonymised with trial number allocations. Data will be recorded on paper case report forms in booklets comprised of triplicate carbon copy paper. The data will be analysed by the clinical research fellow, the chief investigator and the trial statistician. This will primarily be performed at St Thomas' Hospital. This is an 18-month trial (total 3 months of follow-up) with subsequent data analysis and manuscript production. The VAS Score will be measured by two members of the trial team at the coordinating centre. Case Report Forms will be stored in locked filing cabinets. The completed case reports forms will undergo quality control assessment once the patient has completed or withdrawn from the trial by the trial coordinator and manager before transcription onto a password-protected database on a secure computer network. This will performed by two members of the trial team to ensure quality control and data reliability. As this is a non-CTIMP trial, a data monitoring committee is not required; however, the interim analysis will be performed by the trial statistician independent of the investigators.

### Adverse event reporting

Any serious adverse event (SAE) related to the study procedures or is an unexpected occurrence (ie, the type of event is not listed in the protocol as an expected occurrence) must be reported immediately on knowledge of the event to the sponsor within 24 hours. All other AEs must be reported to the sponsor when copied into the annual progress report. Given this is a multisite trial, the principal investigators at all sites must report all SAEs to the chief investigator first where possible. The chief investigator is responsible for reporting events to the sponsor.

#### Expected AEs

These events are expected based on what is already documented for events associated with the trial interventions. The following are considered to be expected AEs associated with the proposed interventions for this trial:

Chest drain and IPC insertion:

1. Pain at drain site
2. Localised bleeding at drain site
3. Localised infection at the drain site
4. Empyema
5. Pneumothorax

Talc pleurodesis

1. Pain
2. Fever

Other expected AEs

1. Death secondary to underlying malignancy
2. Hospitalisation due to underlying malignancy or comorbid condition

These are well-acknowledged risks, and patients will be consented appropriately. Participant safety will be ensured through regular review and follow-up. During the procedures, vital signs including blood pressure and oxygen saturation measurements will be used to ensure patient well-being. Chest X-ray will be used to verify tube position.

## Ethics, approvals and dissemination

Regional Ethics Committee approval was granted by the NRES Committee South East Coast—Brighton and Sussex on the 22 June 2015 (reference 15/LO/1018).

Any important protocol modifications (such as changes to the eligibility criteria, outcomes and analyses) that have been approved by the Health Research Authority will be distributed to all principal investigators and trial team members at each site. These will also be communicated to the trial registries as well as journals at the time of submission.

Approval was granted by the European Organisation for Research and Treatment of Cancer for use of the EORTC QLQ-C30 questionnaire.

We will disseminate the results regardless of outcome. These will be broadcast to key stakeholders through conference presentations and peer review journal publications.

## Discussion

In the decision-making process, the impact of MPE on health-related QoL, type and stage of underlying cancer, performance status, prognosis and patient preference should be considered. Part of the difficulty in evaluating the comparative effectiveness of MPE treatments relates to how outcomes are defined in previous studies. QoL assessments are infrequent and often not performed with validated tools.

We outline the protocol and design of the Out Patient Talc Slurry via Indwelling Pleural Catheter for Malignant Pleural Effusion vs Usual Inpatient Management (OPTIMUM) study. With a recruitment target of 142 patients, this multicentre randomised controlled trial is designed to detect a meaningful difference in global health status outcomes and will provide key comparable data on health-related QoL outcomes using an outpatient ambulatory pathway versus inpatient chest drain insertion and pleurodesis in the management of MPE.

## Footnotes

i Histocytological evidence of pleural malignancy. If a histocytological diagnosis cannot be obtained, clinical correlation combined with radiological features and primary tumour histology can be used to make the diagnosis.

ii Lymphoma and small cell carcinoma are particularly sensitive to treatment with chemotherapeutic agents. If patients have undergone chemotherapy with no treatment response or deemed not for chemotherapy and for palliative management, then they will be suitable for inclusion in the study. Liason with the patient's oncologist or multi-disciplinary team discussion will be required to ascertain this.

## References

[1] Muduly D, Deo S, Subi Ts An update in the management of malignant pleural effusion. Indian J Palliat Care 2011;17:98–103. 10.4103/0973-1075.84529 21976848 PMC3183615

[2] Rahman NM, Ali NJ, Brown G Local anaesthetic thoracoscopy: British Thoracic Society pleural disease guideline 2010. Thorax 2010;65(Suppl 2):ii54–60. 10.1136/thx.2010.137018 20696694

[3] Clive AO, Kahan BC, Hooper CE Predicting survival in malignant pleural effusion: development and validation of the LENT prognostic score. Thorax 2014;69:1098–104. 10.1136/thoraxjnl-2014-205285 25100651 PMC4251306

[4] Roberts ME, Neville E, Berrisford RG Management of a malignant pleural effusion: British Thoracic Society pleural disease guideline 2010. Thorax 2010;65(Suppl 2):ii32–40. 10.1136/thx.2010.136994 20696691

[5] Davies HE, Mishra EK, Kahan BC Effect of an indwelling pleural catheter vs chest tube and talc pleurodesis for relieving dyspnea in patients with malignant pleural effusion: the TIME2 randomized controlled trial. JAMA 2012;307:2383–9. 10.1001/jama.2012.5535 22610520

[6] Musani AI, Haas AR, Seijo L Outpatient management of malignant pleural effusions with small-bore, tunneled pleural catheters. Respiration 2004;71:559–66. 10.1159/000081755 15627865

[7] Ahmed L, Ip H, Rao D Talc pleurodesis through indwelling pleural catheters for malignant pleural effusions: retrospective case series of a novel clinical pathway. Chest 2014;146:e190–4. 10.1378/chest.14-0394 25451360

[8] Harris RJ, Kavuru MS, Mehta AC The impact of thoracoscopy on the management of pleural disease. Chest 1995;107:845–52. 10.1378/chest.107.3.845 7874962

[9] Viallat JR, Rey F, Astoul P Thoracoscopic talc poudrage pleurodesis for malignant effusions. A review of 360 cases. Chest 1996;110:1387–93. 8989050 10.1378/chest.110.6.1387

[10] Shaw P, Agarwal R Pleurodesis for malignant pleural effusions. Cochrane Database Syst Rev 2004;(1):CD002916 10.1002/14651858.CD002916.pub2 14973997

[11] Putnam JB, Walsh GL, Swisher SG Outpatient management of malignant pleural effusion by a chronic indwelling pleural catheter. Ann Thorac Surg 2000;69:369–75. 10.1016/S0003-4975(99)01482-4 10735665

[12] Tremblay A, Michaud G Single-center experience with 250 tunnelled pleural catheter insertions for malignant pleural effusion. CHEST J 2006;129:362–8. 10.1378/chest.129.2.362 16478853

[13] Schniewind B, Rose T, Woltmann N Clinical outcomes and health-related quality of life after thoracoscopic talc pleurodesis. J Palliat Med 2012;15:37–42. 10.1089/jpm.2011.0149 22248257

[14] King MT The interpretation of scores from the EORTC quality of life questionnaire QLQ-C30. Qual Life Res 1996;5:555–67. 10.1007/BF00439229 8993101

[15] Scott N, Fayers P, Aaronson N EORTC QLQ-C30. Reference values. Brussels: EORTC, 2008.

