# Correction

**DOI:** 10.1136/bmjopen-2016-012795corr1

**Published:** 2016-11-14

Sivakumar P, Douiri A, West A, *et al.* OPTIMUM: a protocol for a multicentre randomised controlled trial comparing Out Patient Talc slurry via Indwelling pleural catheter for Malignant pleural effusion vs Usual inpatient Management. *BMJ Open* 2016;6:10:e012795.

In the ‘Inclusion criteria’ section, the bullet point: “Expected survival <3 months” should read “Expected survival >3 months”.

